# Performance of three multi-species rapid diagnostic tests for diagnosis of *Plasmodium falciparum *and *Plasmodium vivax *malaria in Oromia Regional State, Ethiopia

**DOI:** 10.1186/1475-2875-9-297

**Published:** 2010-10-27

**Authors:** Ruth A Ashton, Takele Kefyalew, Gezahegn Tesfaye, Helen Counihan, Damtew Yadeta, Bonnie Cundill, Richard Reithinger, Jan H Kolaczinski

**Affiliations:** 1 Malaria Consortium - Ethiopia Office, Ethio-China Road, PO Box 100224, Addis Ababa, Ethiopia; 2 Malaria Consortium - Africa Regional Office, PO Box 8045, Plot 2, Sturrock Road, Kampala, Uganda; 3 Malaria Consortium International, Development House 56-64, Leonard Street, London, EC2A, 4LT, UK; 4 Oromia Regional Health Bureau, PO Box 24341, Addis Ababa, Ethiopia; 5 London School of Hygiene & Tropical Medicine, Keppel Street, London, UK; 6 U.S. Agency for International Development, Addis Ababa, Ethiopia

## Abstract

**Background:**

Malaria transmission in Ethiopia is unstable and variable, caused by both *Plasmodium falciparum *and *Plasmodium vivax*. The Federal Ministry of Health (FMoH) is scaling up parasitological diagnosis of malaria at all levels of the health system; at peripheral health facilities this will be through use of rapid diagnostic tests (RDTs). The present study compared three RDT products to provide the FMoH with evidence to guide appropriate product selection.

**Methods:**

Performance of three multi-species (pf-HRP2/pan-pLDH and pf-HRP2/aldolase) RDTs (CareStart^®^, ParaScreen^® ^and ICT Combo^®^) was compared with 'gold standard' microscopy at three health centres in Jimma zone, Oromia Regional State. Ease of RDT use by health extension workers was assessed at community health posts. RDT heat stability was tested in a controlled laboratory setting according to WHO procedures.

**Results:**

A total of 2,383 patients with suspected malaria were enrolled between May and July 2009, 23.2% of whom were found to be infected with *Plasmodium *parasites by microscopy. All three RDTs were equally sensitive in detecting *P. falciparum *or mixed infection: 85.6% (95% confidence interval 81.2-89.4). RDT specificity was similar for detection of *P. falciparum *or mixed infection at around 92%. For detecting *P. vivax *infection, all three RDTs had similar sensitivity in the range of 82.5 to 85.0%. CareStart had higher specificity in detecting *P. vivax *(97.2%) than both ParaScreen and ICT Combo (p < 0.001 and p = 0.05, respectively). Health extension workers preferred CareStart and ParaScreen to ICT Combo due to the clear labelling of bands on the cassette, while the 'lab in a pack' style of CareStart was the preferred design. ParaScreen and CareStart passed all heat stability testing, while ICT Combo did not perform as well.

**Conclusions:**

CareStart appeared to be the most appropriate option for use at health posts in Ethiopia, considering the combination of quantitative performance, ease of use and heat stability. When new products become available, the choice of multi-species RDT for Ethiopia should be regularly re-evaluated, as it would be desirable to identify a test with higher sensitivity than the ones evaluated here.

## Background

Approximately 52 million people in Ethiopia are considered to be at risk of malaria [1]. The disease is reportedly the leading cause of morbidity, accounting for about 12% of the total outpatient visits and 9.9% of the total admissions in 2007 [2]. Between 4-6 million clinical malaria cases are reported annually across all health facilities in the country, however the actual number of malaria cases is estimated to be as high as 10-15 million [3]. The major *Plasmodium *species causing malaria in Ethiopia are *Plasmodium falciparum *and *Plasmodium vivax*, for which the national guidelines prescribe treatment with the artemisinin combination therapy (ACT) artemether-lumefantrine (CoArtem^®^) or with chloroquine, respectively; *Plasmodium ovale *and *Plasmodium malariae *are rare. To reduce costs and minimise selection for drug resistance in *Plasmodium *parasites there is considerable pressure to minimise ACT use. This can only be achieved once parasitological diagnosis of malaria is routinely provided at all levels of the health system. Parasitological diagnosis is particularly important in low transmission settings where a large proportion of febrile illness is due to causes other than malaria.

Rapid diagnostic tests (RDTs) for detection of *P. falciparum *(ParaCheck-Pf^®^, Orchid Biomedical Systems, Goa, India) have been used by health extension workers (HEWs) at health posts in Ethiopia since 2005 [3-7]. (Description of Ethiopian health system in Additional file 1). Currently, there is no capacity for parasitological diagnosis of non-falciparum malaria at health facilities without laboratory services. RDTs using *Plasmodium *lactate dehydrogenase (pLDH) to detect non-falciparum malaria were initially less reliable than histidine-rich protein 2 (HRP2)-based tests [8-11]. However, recent studies have shown sensitivity and specificity of pLDH-based RDTs approaching those of falciparum-only tests [12-15], to the extent where they may be appropriate for routine use in case management at facilities where microscopy is not available.

The present study was conducted to determine the performance of three multi-species RDTs for diagnosis of *P. falciparum *and *P. vivax *malaria in Ethiopia. The aim of this study was to establish which multi-species RDTs can be recommended for routine use at health posts in Ethiopia.

## Methods

### Study setting

**Figure 1:**
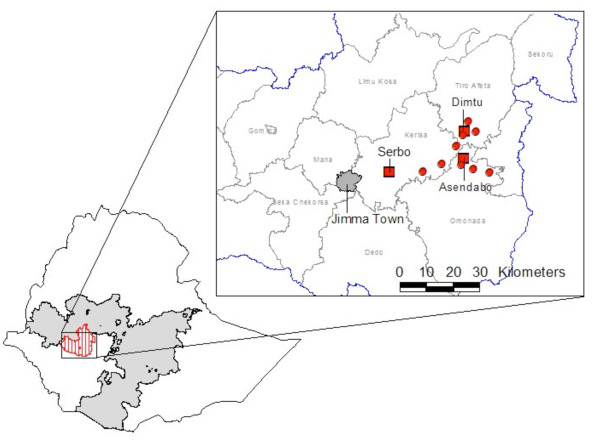
**Location of Oromia Regional State (solid shading) and Jimma zone (vertical shading) within Ethiopia**. Detail of Jimma zone indicates study health centres (filled squares) and health posts (filled circles).

The study was conducted from April to August 2009 in three woredas; Omo Nada, Kersa and Tiro Afeta; in the Jimma zone of Oromia Regional State, Ethiopia (Figure 1). Within each woreda, one health centre and three health posts were purposively selected according to reported malaria cases. Jimma zone is 300 km south-west of Addis Ababa, and has an elevation of approximately 1,780 meters above sea level. Malaria transmission in Oromia is perennial, but peaks from April to May and from October to December, after the seasonal rains.

### Rapid Diagnostic Tests

Three RDT products were compared: i) CareStart^® ^pf-HRP2/pan-pLDH (AccessBio, USA, catalogue number G0131SK), ii) ParaScreen^® ^pf-HRP2/pan-pLDH (Zephyr Biomedicals, India, catalogue number 50310025), and iii) ICT Combo^® ^pf-HRP2/pan-aldolase (ICT Diagnostics, South Africa, catalogue number ML02). All of the respective RDTs were from the same lot, with expiry date July 2010 (CareStart) or January 2011 (ParaScreen and ICT Combo). CareStart RDTs are individually packaged with swab, lancet, capillary tube, and buffer. ParaScreen cassettes and sample loop are individually packaged, with a single buffer bottle per box. ICT Combo cassettes are individually wrapped but buffer and all other components are provided separately in the box.

### Health centre quantitative assessment

Procedures were developed in accordance with Standards for Reporting of Diagnostic Accuracy (STARD) guidelines [16]. Patients attending the outpatient department of the three health centres with symptoms of uncomplicated malaria (axillary temperature > 37.5°C or report of fever in the previous 48 hours) were recruited into the study. Patients under six months of age or with any life-threatening condition were excluded. RDTs were transported unrefrigerated by air to Addis Ababa, where they were stored at ambient conditions until transfer to field sites. Temperature was monitored (Tinytag, Gemini Data Loggers, UK) but not controlled while RDTs were transported by road to health centres and during storage at the health centres. Temperatures during transport reached a maximum 36°C, but at health facilities temperatures did not exceed 30°C.

Sample size was calculated to compare performance of each RDT to microscopy in detecting both *P. falciparum *and *P. vivax *at each site, assuming RDT sensitivity of ≥ 95% and specificity of ≥ 80%, with 80% power and a 5% significance level [17]. Assuming > 15% of febrile patients have microscopy-confirmed malaria infection, at an estimated 60:40 proportion of *P. falciparum *to *P. vivax*, a total of 811 febrile patients were estimated to be needed at each of the three sites.

Basic demographic information and clinical details were recorded from each enrolled patient. A single finger prick was performed by a laboratory technician or nurse, and used to prepare two slides, each with one thick and one thin film. The same finger prick blood sample was used to carry out all three RDTs in parallel, following manufacturer's instructions. Thin blood films were fixed in methanol after air-drying, then slides were stained in 10% Giemsa solution for 15 minutes. Thick films were read at the health centre by a laboratory technician and considered negative if no parasites were seen after examination of 200 fields at ×1,000 magnification. When positive for parasites, the number of asexual parasites per 200 white blood cells, or 500 white blood cells for low density infections, were used to calculate the number of asexual parasites per μl of blood, assuming a standard count of 8,000 white blood cells per μl of blood [18]. Species, number of parasites/μl (p/μl) and presence of gametocytes were recorded. RDT and microscopy results were read by different staff at the health centre, each blinded to the results of the other diagnostic technique.

All blood films were re-read a second time by an experienced microscopist at a regional malaria reference laboratory, blinded to initial microscopy and RDT results. A third, blinded, reading was conducted on all slides with discrepant first and second readings: presence/absence of asexual parasites, difference in species, or > 50% difference in parasite count. Microscopy results and parasite counts were corrected according to the third reading.

### Health post ease of use assessment

Each health post was staffed by two female HEWs or nurses, who received basic training on the use and interpretation of multi-species RDTs. Each carried out 50 tests with each RDT product during their normal duties both at the health post and during community outreach visits. RDTs were discarded after use. Any patient with suspected malaria was included in the sample.

Interviews were conducted with HEWs after completion of each set of 50 RDTs. Respondents were asked to grade eleven specific features of the test, such as the packaging, blood collection device, buffer and results interpretation (Additional file 2), followed by open-ended questions to probe for any further preferences. The various aspects of each RDT were graded on a scale of one (difficult) to five (very easy). After completion of all three RDT products, a final interview round was conducted to assess comparative preferences.

### Heat stability assessment

Heat stability assessments were conducted between August and December 2009 at the Ethiopian Health and Nutrition Research Institute located in Addis Ababa, designated as a regional reference laboratory by the World Health Organization (WHO) and the Foundation for Innovative New Diagnostics (FIND).

Heat stability and lot testing of RDTs was conducted according to the standard protocol developed by WHO/FIND [19,20]. RDTs were exposed to 35°C and 45°C for up to 90 days and to 60°C for up to 72 hours. At pre-determined periods, RDTs were removed from incubators and allowed to reach room temperature, then HRP2 and pLDH or aldolase bands were assessed individually with a negative control and using cultured *P. falciparum *and *P. vivax *at concentrations of 200 and 2,000 p/μl. For the low parasite density infection, two of each RDT were tested because of the expected lowered sensitivity, while only one of each RDT was tested at each time point with high parasite density samples.

### Statistical analysis

Health centre data were entered and verified using Microsoft^® ^Access 2007 (Microsoft Corporation, Seattle). Second and third blood film microscopy results were entered into a Microsoft^® ^Excel 2007 spreadsheet (Microsoft Corporation, Seattle). All data were analyzed using STATA version 8.0 (STATA Corporation, College Station, TX, USA). RDT performance was assessed in three categories: negative, non-falciparum mono-infection, and either mixed infection with *P. falciparum *and a non-falciparum species, or *P. falciparum *mono-infection. The sensitivity, specificity, negative predictive value (NPV) and positive predictive value (PPV) were calculated with 95% confidence intervals (CI) for each RDT [21]. An RDT was categorized as a true positive for *P. vivax *infection if only the pan band showed, while an RDT was categorized as a true negative if the test itself did not show either of the two test bands and if the corresponding blood slide was found to be negative for all *Plasmodium *spp. by microscopy. Similarly, an RDT was categorized as a true positive for *P. falciparum *or mixed infection if it showed either the HRP2 and pan band, or HRP2 band only, while it was considered as a true negative if it showed no test bands and if no *Plasmodium *spp. infection was diagnosed by examination of the corresponding blood slide using microscopy. The Kappa coefficient (κ), representing the proportion of agreements beyond chance, was used to quantify the level of agreement between each RDT and 'gold standard' microscopy; κ ≥ 0.8 was considered to indicate high reliability [22]. No adjustments were made for multiple comparison testing. Each of these performance indicators were compared between two RDTs using McNemar's test [23] to account for the paired nature of results.

For each of the eleven specific features of the RDTs graded by HEWs the mean score was calculated and each test received an overall rating: RDTs were ranked according to their combined score. All responses to open-ended questions given during interviews were read and coded according to common features.

### Ethical considerations

The study protocol was approved by the institutional review boards of the London School of Hygiene and Tropical Medicine (Application No. 5444) and the Ethiopian Science and Technology Agency. Patients (or guardians of children ≤ 16 years) recruited at health centres provided written informed consent prior to inclusion. Study participants with microscopy-confirmed malaria infection were treated according to national guidelines [24]. At health posts, patients provided verbal consent before testing with a multi-species RDT was conducted in addition to routine ParaCheck use.

## Results

### Study population description

A total of 2,400 febrile patients were enrolled into the study between May 1 and July 31 2009, 800 at each of the three health centres. Data from 17 individuals were excluded due to missing RDT results or unreadable blood films, therefore full data are available from 2,383 individuals.

### Microscopy results

**Figure 2:**
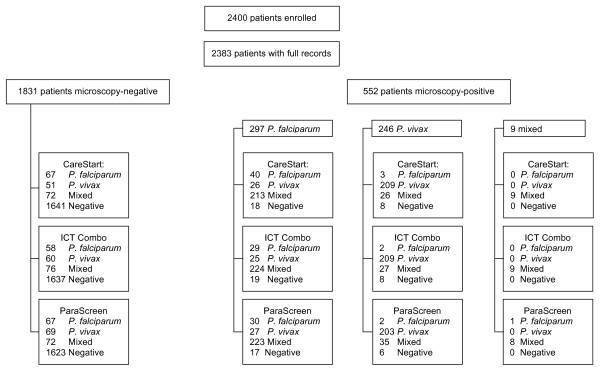
**Flowchart of microscopy and RDT results**.

**Figure 3:**
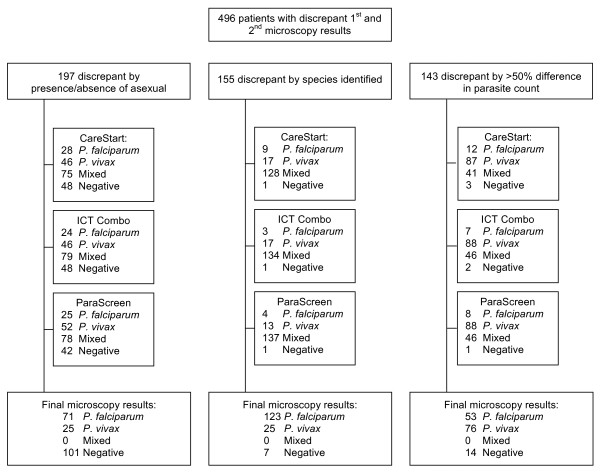
**Flowchart of microscopy results discrepant between first and second readings**.

Five hundred and fifty two (23.2%) patients were diagnosed with malaria by microscopy, of which (297) 53.8% and (246) 44.6% were infected with *P. falciparum *and *P. vivax *respectively. Nine (1.6%) confirmed cases had mixed infection of *P. falciparum *and *P. vivax *(Figure 2). Eight individuals had gametocytes (seven *P. falciparum*, one *P. vivax) *but no asexual parasites. All 2,383 blood slides were read twice. A total of 496 slides were read a third time to address discrepancies following the first and second microscopy readings (Figure 3).

One third (38.4%) of *P. falciparum *cases had parasite densities > 5,000 p/μl and one-fifth (20.6%) < 200 p/μl. About half (52.6%) of patients with *P. vivax *had parasite density > 5,000 p/μl, while only 3.5% had low density infection (< 200 p/μl). The geometric mean (95% CI) parasite count was 1,681 p/μl (1315-2148) for *P. falciparum *and 3,406 p/μl (2844-4080) for *P. vivax*.

### RDT compared to 'gold standard' microscopy

RDT results had moderate agreement with microscopy in detecting *P. falciparum *or mixed infection (κ range 0.6910 to 0.6976) and moderate to good agreement with microscopy for detection of non-falciparum malaria (κ range 0.7531 to 0.8020) (Table 1).

**Table 1:** Comparative performance indicators of each RDT

	CareStart	ParaScreen	ICT Combo	**McNemar p value**^**1**^		
***P. falciparum^2^***	n = 2137	n = 2137	n = 2137	CS-PS	CS-ICT	ICT-PS

Sensitivity (95% CI^3^)	85.6% (81.2-89.4)	85.6% (81.2-89.4)	85.6% (81.2-89.4)	1.0	1.0	1.0
Specificity (95% CI)	92.4% (91.1-93.6)	92.4% (91.1-93.6)	92.7% (91.4-93.8)	1.0	0.10	0.10
PPV (95% CI)	65.3% (60.5-70.0)	65.3% (60.5-70.0)	66.2% (61.3-70.8)	1.0	0.76	0.76
NPV (95% CI)	97.5% (96.6-98.2)	97.5% (96.6-98.2)	97.5% (96.6-98.2)	1.0	1.0	1.0
κ^4^	0.6910	0.6910	0.6976	1.0	0.70	0.70

**Non-falciparum only^5^**	n = 2077	n = 2077	n = 2077	CS-PS	CS-ICT	ICT-PS

Sensitivity (95% CI)	85.0% (79.9-89.2)	82.5% (77.2-87.1)	85.0% (79.9-89.2)	0.11	1.0	0.08
Specificity (95% CI)	97.2% (96.4-97.9)	96.2% (95.3-97.1)	96.7% (95.8-97.5)	**< 0.001**	**0.05**	0.11
PPV (95% CI)	80.4% (75.0-85.0)	74.7% (69.0-79.7)	77.7% (72.2-82.6)	0.10	0.39	0.43
NPV (95% CI)	98.0% (97.2-98.6)	97.6% (96.8-98.3)	98.0% (97.2-98.6)	0.78	1.0	0.50
κ	0.8020	0.7531	0.7851	**< 0.001**	0.23	**0.03**

^1 ^Significant relationships (p > 0.05) in bold type

^2 ^Individuals with *P. falciparum *mono-infection or mixed infection with *P. falciparum *and *P. vivax*. *P. vivax *mono-infection excluded.

^3 ^95% Confidence interval

^4 ^Kappa (κ) is the proportion agreement between microscopy and RDT results, where 0.8 indicates good agreement.

^5 ^Individuals with *P. vivax *mono-infection. Mixed infections and *P. falciparum *mono-infection excluded.

For some *Plasmodium*-infected patients the RDT result was positive but the species was incorrectly identified. The most common species misdiagnosis was where the RDT showed only the Pan band for *P. falciparum *mono-infections (26 CareStart, 27 ParaScreen and 25 ICT Combo). Only two of these infections were > 200 p/μl; with 12,800 p/μl and 48,000 p/μl. Additionally, RDTs occasionally indicated *P. falciparum *mono-infection for what was diagnosed by microscopy to be *P. vivax *mono-infection (three CareStart, two ParaScreen and two ICT Combo). Interestingly, all nine mixed infections resulted in HRP2 and pan band activation in CareStart and ICT Combo, while the ParaScreen pan band failed to show in one of the nine mixed infections.

The sensitivity of all RDTs in detecting *P. falciparum *infections was 85.6%. Specificity was statistically similar for all three RDTs at 92.4% to 92.7% (Table 1). The PPVs for the RDTs were comparable in detecting *P. falciparum *infection at around 65%. NPVs were the same for all RDTs at 97.5%. The false negative RDT results for *P. falciparum *had parasite density between 40 and 50,000 p/μl, with median density 80 p/μl. Species misdiagnoses were not considered as false negative results.

CareStart was the best performing RDT in detecting non-falciparum infections based on kappa (κ = 0.8020) (Table 1), with both CareStart and ICT Combo showing superior kappa values to that of ParaScreen (p < 0.001 and p = 0.03, respectively). The PPV and NPV of all three RDTs were comparable (p > 0.05 for all pairs), however specificity of CareStart was significantly higher than that of ParaScreen and ICT Combo (p < 0.001 and p = 0.05, respectively). False negative RDT results against *P. vivax *were generally low density infections: range 40 to 7080 p/μl with median 280 p/μl. Few of the false positive RDT results for *P. vivax *or *P. falciparum *were due to residual antigen from cleared infections, since only 11% of patients with false positive results had received anti-malaria treatment in the previous four weeks.

Sensitivity was calculated for *P. falciparum *infection and for *P. vivax *mono-infection at different parasite densities (< 200 p/μl of blood; 200-500 p/μl; 500-5,000 p/μl; > 5,000 p/μl) (Table 2). All three RDTs showed improved sensitivity with increasing *P. falciparum *density (p < 0.001), but sensitivity was generally comparable between RDTs within each parasite density category. No statistical association was seen between *P. vivax *density and RDT sensitivity, likely due to the small number of low density infections. However, CareStart and ICT Combo were seen to be more sensitive than ParaScreen in detection of *P. vivax *infection at > 5000 p/μl (p = 0.03).

**Table 2:** Sensitivities of RDTs at different parasite densities

		CareStart	ParaScreen	ICT Combo	**McNemar p value**^**1**^		
*P. falciparum*							
Parasite density	N	Sensitivity: Pf or Pf+pan^2 ^(95% CI^3^)			CS-PS	CS-ICT	ICT-PS

< 200 p/μl	63	66.7% (53.7-78.1)	66.7% (53.7-78.1)	66.7% (53.7-78.1)	1.0	1.0	1.0
< 500 p/μl	90	70.0% (59.4-79.2)	70.0% (59.4-79.2)	70.0% (59.4-79.2)	1.0	1.0	1.0
< 5000 p/μl	188	79.1% (72.6-84.7)	79.1% (72.6-84.7)	79.1% (72.6-84.7)	1.0	1.0	1.0
> 5000 p/μl	118	95.8% (90.4-98.6)	95.7% (90.4-98.6)	95.7% (90.4-98.6)	1.0	1.0	1.0

*P. vivax*							
Parasite density	N	Sensitivity: pan band only^4 ^(95% CI)			CS-PS	CS-ICT	ICT-PS

< 200 p/μl	9	77.8% (40.0-97.2)	88.9% (51.8-99.7)	66.7% (29.9-92.5)	0.32	0.56	0.16
< 500 p/μl	28	82.1% (63.1-93.9)	89.3% (71.8-97.7)	85.7% (67.3-96.0)	0.32	0.65	0.56
< 5000 p/μl	116	82.8% (74.6-89.1)	82.8% (74.6-89.1)	82.8% (74.6-89.1)	1.0	1.0	1.0
> 5000 p/μl	128	86.7% (79.6-92.1)	82.8% (75.1-88.9)	86.7% (79.6-92.1)	**0.03**	1.0	**0.03**

^1 ^Significant relationships (p > 0.05) in bold type.

^2 ^RDT result showed either *P. falciparum *HRP2 band only, or HRP2 band and pan pLDH band. Individuals with *P. vivax *infection only excluded.

^3 ^95% Confidence Interval

^4 ^RDT result showing pan band only. Individuals with any *P. falciparum *infection excluded.

### Ease of use assessment

All HEWs involved in the ease of use assessment had previously used ParaCheck, and commented on the importance of using a multi-species RDT in their work at the health post and in the community. *"This kind of multi-species RDT is important for our routine activities and should be immediately applied in our work."*

When all scored components were averaged, CareStart scored highest (4.5) followed by ParaScreen (4.4) and ICT Como (4.2). A number of HEWs indicated that they preferred the packaging of CareStart in individual 'lab in a pack' sets with all components needed for a single test (cassette, swab, lancet, blood collection pipette, buffer, and instructions) included in each packet: "*I liked having all materials within a single packet, it makes moving from house-to-house easier as I don't have to carry a large box, so is very comfortable for my work in the community."*

The most salient factor influencing the ease of use of each RDT was the presence of clear markings on the cassette for interpretation of results. ParaScreen had the most detailed labelling with the band positions marked with 'C' at the control region, then 'Pan' and 'Pf' in the test region. CareStart has 'C' at the control band, with '1' for *P. falciparum *and '2' for Pan. ICT Combo has no separate markings for Pf and Pan bands, meaning that users must remember the band location when reading results.

### Heat stability

CareStart and ParaScreen were shown to have superior stability to that of ICT Combo during the heat stability testing. When tested after more than four hours storage at 60°C, the HRP2 band of ICT Combo failed to detect infection. The ICT Combo HRP2 band showed only very weak positive results when tested after 30 days storage at 35°C and 45°C. However, both CareStart and ParaScreen HRP2 bands were stable after storage for 72 hours at 60°C, and 90 days at 35°C and 45°C, all giving positive results when tested with *P. falciparum*.

All RDTs had pan bands that were more heat-sensitive than HRP2, appearing faint throughout the testing with both high and low density *P. vivax *infections. Both CareStart and ParaScreen pLDH bands completed the testing procedure at 35°C, 45°C and 60°C. The ICT Combo aldolase band failed to detect *P. vivax *at 200 p/μl after 60 days stored at 35°C and after 60 days stored at 45°C. However, after 60 days storage at these temperatures, ICT Combo did detect *P. vivax *at the higher density of 2,000 p/μl. ICT Combo aldolase band failed to detect 200 p/μl *P. vivax *after 72 hours stored at 60°C.

## Discussion

RDTs for malaria are being increasingly adopted across endemic countries to strengthen parasitological diagnosis and appropriate management of all fever cases [25]. A large number of products are now available, and while a detailed assessment of performance has been undertaken by WHO/FIND [19,20] and a list of recommended products produced [26] it is important that the most appropriate products for each transmission setting and operational context are identified. An appropriate RDT for implementation across Ethiopia should be able to detect *P. falciparum *and *P. vivax*, be highly sensitive and specific, and also able to detect low parasite density infections. PPV and NPV give an indication of usefulness of the test in practice, but these indicators are influenced by the prevalence of malaria in the test population. To guide product selection by the FMoH, the present study set out to determine the performance of three multi-species RDTs for diagnosis of *P. falciparum *and *P. vivax *malaria in Ethiopia.

The sensitivity of the three RDTs in detecting *P. falciparum *was found to be similar to their sensitivity in detecting *P. vivax*, and there was little difference in RDT performance when compared to microscopy. Overall, ICT Combo and CareStart showed better performance than ParaScreen. It was unexpected that the RDTs would have similar sensitivity for detection of both *P. falciparum *and *P. vivax *infection, since other studies in co-endemic areas have consistently found multi-species RDTs to be better at detecting *P. falciparum *infection [8,13,27]. The present finding may be due to misdiagnosis and/or under diagnosis of species at low parasite density by microscopy. More low density *P. falciparum *than *P. vivax *infections were detected by microscopy, contrary to findings in other co-endemic areas where *P. falciparum *is usually presents with higher parasite density than *P. vivax *[9,13]. However, a study in Columbia found similar *P. falciparum *and *P. vivax *infection densities, but amongst children under five years the *P. vivax *parasite density was higher than *P. falciparum *[10]. In the present study all slides were subject to rigorous quality control testing: read twice by qualified technicians, with any discrepancies corrected according to an expert microscopist, suggesting that the result is unlikely to be due to human error.

The maximum recommended storage temperatures for CareStart and ParaScreen (30°C) was exceeded in the current study during transport of RDTs to the study sites (maximum temperature during transit 36°C). This is unlikely to have contributed to any reduction in performance since heat stability testing indicated that both CareStart and ParaScreen were able to detect infection after longer periods being stored at higher temperatures. Nevertheless, it is important to consider transport and storage conditions for RDTs during routine use in the health system.

The RDTs had high NPVs, meaning that they were reliable in ruling out malaria. However the lower PPV means that patients will occasionally be falsely diagnosed as positive for malaria and unnecessarily treated. False positives in this study were not due to residual antigen from previous infections, since only 11% of the total false positive results were from patients who had received any anti-malarial treatment in the previous four weeks. Alternative explanations include sequestration: erythrocytes containing mature parasites clump together in the microvasculature, therefore are not seen in the peripheral circulation and blood films, while antigen continues to be released [28]. It may also be possible that the parasite density was too low to be seen by microscopy, but there was sufficient parasite antigen to result in a positive RDT [29]. Whilst previous work has shown that some RDT false positives are due to patients with residual gametocytaemia [30], this association was not seen in the current study.

The WHO/FIND product testing of malaria RDTs [19,20] is the most comprehensive assessment of malaria RDTs on the market to date. Contrary to the present results, ParaScreen performed poorly in the WHO/FIND testing. However CareStart performed slightly better in the WHO/FIND testing than in the current study, as did the HRP2 band of ICT Combo. The aldolase band of ICT Combo was comparatively better at detecting low density *P. vivax *in our findings than in the WHO/FIND tests. There are a number of possible reasons for this differential performance of the same RDTs in the two studies. Firstly, the WHO/FIND product testing was carried out by highly experienced technicians in a controlled laboratory setting, while this study involved staff engaged in routine activities at the health facility. Other influencing factors may have been variation between product lots, the impact of transportation and storage conditions, or the use of fresh finger-prick blood samples from patients rather than previously frozen samples. It is also possible that genetic variation of target antigens can lead to variation in performance of RDTs in different regions: Baker *et al *found significant genetic variation in HRP2 isolates from different countries which was associated with reduced sensitivity of RDTs at low density infections [31]. The prozone effect, whereby immunological tests are not activated by very high density infections, has been suggested as a reason for HRP2 false negative results in malaria RDTs [32]. However in the current study, none of the samples which elicited false-negative RDT results were hyperparasitaemic (> 5% red blood cells parasitised), therefore the prozone effect is unlikely to be contributing to the presence of false negative HRP2 results.

In the Ethiopian context, a number of studies have been carried out to assess performance of a variety of multi-species malaria RDTs, to guide the FMOH decision of a replacement for ParaCheck-Pf. An initial study found ParaScreen to have much lower sensitivity [27] than the present study, while a subsequent investigation during peak transmission season reported comparable performance [33]. A CareStart pf-HRP2/pv-pLDH combination RDT has also been evaluated at two locations in Ethiopia [4,5]. The sensitivity of the test in detecting *P. falciparum *by HRP2 at Wondo Genet was 99.4% and 96.4% in Jimma, both very much higher than for CareStart pf/pan in the current study. The *P. vivax*-specific pLDH band of the pf/pv RDT also appeared to have much higher sensitivity than the Plasmodium-specific pLDH band of the pf/pan RDT tested in this study. Although *P. ovale *and *P. malariae *are believed to be responsible for < 1% of all malaria cases in Ethiopia, there remains a need to provide parasitological diagnosis for these species. It is likely that lower performance of pf/pan RDTs in this study compared to prior pf/pv RDT findings is a result of conducting the study under operational conditions. Furthermore, the WHO/FIND evaluation indicates that pf/pan and pf/pv RDTs have comparable performance [19,20].

CareStart also manufactures a pf-pLDH/pan-pLDH combination test, which has been evaluated in Madagascar [12] and Myanmar [9]. The sensitivity of pan-pLDH in the current study falls between the values determined in Madagascar and Myanmar, indicating that our results for CareStart pan-pLDH are reliable.

Ability of the end-user to correctly prepare and interpret the RDT is crucial to operational success. Ease of use has been shown to vary considerably with different RDT formats [34,35] and reduce test accuracy if health workers do not receive sufficient training or support tools [36]. WHO acknowledges the importance of selecting an RDT appropriate to the level of training and supervision of the end-user [19]. The 'lab in a pack' format and clear labelling of the cassette bands were factors that HEWs valued, supporting adoption of CareStart for use at health posts in Ethiopia, but the current work did not investigate whether HEW preferences affect the use of RDT results to make case management decisions. In order for multi-species RDTs to be fully effective at health post level, it is necessary to confirm that results of RDTs are correctly transferred to treatment or referral procedures.

Results of heat stability assessment for CareStart were consistent between the WHO/FIND testing and the current study, with strong detection rates after storage at 35°C and 45°for low and high-density *P. falciparum *and *P. vivax *samples. ParaScreen was found to perform better in heat stability testing during the current study than in the WHO/FIND product testing, where failures occurred with low density *P. falciparum *and *P. vivax *infections. ICT Combo performed well in WHO/FIND testing, with the HRP2 band detecting all *P. falciparum *samples, but in our study ICT Combo failed to detect one high density *P. falciparum *sample and showed weak responses towards the end of the testing period. Test procedures were the same for both studies and the discrepancy in ParaScreen results may therefore be due to variation between product lots.

## Conclusions

All three RDTs showed performance below WHO recommendations for sensitivity compared to 'gold standard microscopy'. Health extension workers were easily able to adapt to the use of multi-species RDTs after limited training. For use at health posts, ICT Combo seemed least suitable, as it did not provide reliable results after being stored at ambient temperatures. Considering a combination of reliability compared to microscopy, heat stability, and ease of use judged by health extension workers, CareStart pf-HRP2/pan-pLDH is found to be the most appropriate tool to allow for parasitological diagnosis at health post level in Ethiopia. However the selection of RDT should be regularly re-evaluated as new products are released, to ensure that the most appropriate test is used.

## List of abbreviations

ACT: artemisinin-based combination therapy; FIND: Foundation for Innovative New Diagnostics; FMoH: Federal Ministry of Health; HEW: health extension worker; HRP2: histidine-rich protein-2; NPV: negative predictive value; pLDH: Plasmodium lactate dehydrogenase; PPV: positive predictive value; RDT: rapid diagnostic test; WHO: World Health Organization.

## Competing interests

The authors declare that they have no competing interests.

## Authors' contributions

RA contributed to study design, supervised data management and cleaning, conducted analysis and interpretation of data and drafted the manuscript. TK led training of health centre and health post staff, supervised data collection at health facilities and heat stability testing. GT and DY coordinated field activities and heat stability testing. BC contributed to data analysis and interpretation, and revised the manuscript. HC, RR and JK conceived the study, contributed to study design, data interpretation and revised the manuscript. All authors approved the final manuscript.

## Supplementary Material

Additional file 1 **Description of the Ethiopian health system**. Click here for file

Additional file 2 **Semi-structured questionnaire used to evaluate RDT ease of use by health extension workers**. Click here for file

## References

[B1] Federal Democratic Republic of Ethiopia MoH Guideline for malaria epidemic prevention and control in Ethiopia 2004 2 Addis Ababa, Ethiopia

[B2] Federal Democratic Republic of Ethiopia MoH Health and Health Related Indicators 2007 Addis Ababa, Ethiopia

[B3] Federal Democratic Republic of Ethiopia MoH National Five Year Strategic Plan for Malaria Prevention and Control in Ethiopia 2006 Addis Ababa, Ethiopia

[B4] Mekonnen Z Ali S Belay G Suleman S Chatterjee S Evaluation of the performance of CareStart Malaria Pf/Pv Combo Rapid Diagnostic Test for the diagnosis of malaria in Jimma, southwestern Ethiopia Acta Trop 2010 113 285 288 10.1016/j.actatropica.2009.12.001 20005196

[B5] Sharew B Legesse M Animut A Jima D Medhin G Erko B Evaluation of the performance of CareStart Malaria Pf/Pv Combo and Paracheck Pf tests for the diagnosis of malaria in Wondo Genet, southern Ethiopia Acta Trop 2009 111 321 324 10.1016/j.actatropica.2009.05.014 19482001

[B6] Nigussie D Legesse M Animut A H/Mariam A Mulu A Evaluation of Paracheck pf and Parascreen pan/pf tests for the diagnosis of malaria in an endemic area, South Ethiopia Ethiop Med J 2008 46 375 381 19271402

[B7] Lemma H Byass P Desta A Bosman A Costanzo G Toma L Fottrell E Marrast AC Ambachew Y Getachew A Mulure N Morrone A Bianchi A Barnabas GA Deploying artemether-lumefantrine with rapid testing in Ethiopian communities: impact on malaria morbidity, mortality and healthcare resources Trop Med Int Health 2009 15 241 250 10.1111/j.1365-3156.2009.02447.x 19961564

[B8] Hopkins H Kambale W Kamya MR Staedke SG Dorsey G Rosenthal PJ Comparison of HRP2- and pLDH-based rapid diagnostic tests for malaria with longitudinal follow-up in Kampala, Uganda Am J Trop Med Hyg 2007 76 1092 1097 17556616

[B9] Ashley EA Touabi M Ahrer M Hutagalung R Htun K Luchavez J Dureza C Proux S Leimanis M Lwin MM Koscalova A Comte E Hamade P Page A-L Nosten F Guerin PJ Evaluation of three parasite lactate dehydrogenase-based rapid diagnostic tests for the diagnosis of *falciparum *and *vivax *malaria Malar J 2009 8 241 10.1186/1475-2875-8-241 19860920 PMC2774865

[B10] van den Broek I Hill O Gordillo F Angarita B Hamade P Counihan H Guthmann J-P Evaluation of three rapid tests for diagnosis of *P. falciparum *and *P. vivax *malaria in Colombia Am J Trop Med Hyg 2006 75 1209 1215 17172395

[B11] Pattanasin S Proux S Chompasuk D Luwiradaj K Jacquier P Looareesuwan S Nosten F Evaluation of a new Plasmodium lactate dehydrogenase assay (OptiMAL-IT) for the detection of malaria Trans R Soc Trop Med Hyg 2003 97 672 674 10.1016/S0035-9203(03)80100-1 16117960

[B12] Ratsimbasoa A Randriamanantena A Raherinjafy R Rasoarilalao N Ménard D Which malaria rapid test for Madagascar? Field and laboratory evaluation of three tests and expert microscopy of samples from suspected malaria patients in Madagascar Am J Trop Med Hyg 2007 76 481 485 17360871

[B13] Bharti PK Silawat N Singh PP Singh MP Shukla M Chand G Dash AP Singh N The usefulness of a new rapid diagnostic test, the First Response Malaria Combo (pLDH/HRP2) card test, for malaria diagnosis in the forested belt of central India Malar J 2008 7 126 10.1186/1475-2875-7-126 18620560 PMC2478667

[B14] Fogg C Twesigye R Batwala V Piola P Nabasumba C Kiguli J Mutebi F Hook C Guillerm M Moody A Guthmann J-P Assessment of three new parasite lactate dehydrogenase (pan-pLDH) tests for diagnosis of uncomplicated malaria Trans R Soc Trop Med Hyg 2008 102 25 31 10.1016/j.trstmh.2007.09.014 18031779

[B15] Gerstl S Dunkley S Mukhtar A De Smet M Baker S Maikere J Assessment of two malaria rapid diagnostic tests in children under five years of age, with follow-up of false-positive pLDH test results, in a hyperendemic falciparum malaria area, Sierra Leone Malar J 2010 9 28 10.1186/1475-2875-9-28 20092620 PMC2835716

[B16] Bossuyt PM Reitsma JB Bruns DE Gatsonis CA Glasziou PP Irwig LM Lijmer JG Moher D Rennie D de Vet HC Towards complete and accurate reporting of studies of diagnostic accuracy: the STARD initiative BMJ 2003 326 41 44 10.1136/bmj.326.7379.41 12511463 PMC1124931

[B17] Jones SR Carley S Harrison M An introduction to power and sample size estimation Emerg Med J 2003 20 453 458 10.1136/emj.20.5.453 12954688 PMC1726174

[B18] WHO Basic malaria microscopy 1991 Geneva: World Health Organization

[B19] WHO Malaria rapid diagnostic test performance: results of WHO product testing of malaria RDTs: round 1 2009 Geneva: World Health Organization

[B20] WHO Malaria rapid diagnostic test performance: results of WHO product testing of malaria RDTs: round 2 2010 Geneva: World Health Organization

[B21] WHO Methods for field trials of malaria rapid diagnostic tests 2009 Geneva: World Health Organization

[B22] McGinn T Tips for learners of evidence-based medicine: 3. Measures of observer variability (kappa statistic) CMAJ 2004 171 1369 1373 15557592 10.1503/cmaj.1031981 PMC527344

[B23] Lachenbruch PA Lynch CJ Assessing screening tests: extensions of McNemar's test Stat Med 1998 17 2207 2217 10.1002/(SICI)1097-0258(19981015)17:19<2207::AID-SIM920>3.0.CO;2-Y 9802179

[B24] Federal Democratic Republic of Ethiopia MoH Malaria diagnosis and treatment guidelines for health workers in Ethiopia 2004 Addis Ababa, Ethiopia

[B25] WHO World Malaria Report 2009 2009 Geneva: World Health Organization

[B26] WHO Information note on interim selection criteria for procurement of malaria rapid diagnostic tests (RDTs) 2010 Geneva: World Health Organisation, Global Malaria Programme

[B27] Endeshaw T Gebre T Ngondi J Graves PM Shargie EB Ejigsemahu Y Ayele B Yohannes G Teferi T Messele A Zerihun M Genet A Mosher AW Emerson PM Richards FO Evaluation of light microscopy and rapid diagnostic test for the detection of malaria under operational field conditions: a household survey in Ethiopia Malar J 2008 7 118 10.1186/1475-2875-7-118 18598344 PMC2474640

[B28] Dondorp AM Desakorn V Pongtavornpinyo W Sahassananda D Silamut K Chotivanich K Newton PN Pitisuttithum P Smithyman AM White NJ Day NPJ Estimation of the total parasite biomass in acute falciparum malaria from plasma PfHRP2 PLoS Med 2005 2 e204 10.1371/journal.pmed.0020204 16104831 PMC1188247

[B29] Bell DR Wilson DW Martin LB False-positive results of a *Plasmodium falciparum *histidine-rich protein 2-detecting malaria rapid diagnostic test due to high sensitivity in a community with fluctuating low parasite density Am J Trop Med Hyg 2005 73 199 203 16014858

[B30] Tjitra E Suprianto S McBroom J Currie BJ Anstey NM Persistent ICT malaria P.f/P.v panmalarial and HRP2 antigen reactivity after treatment of *Plasmodium falciparum *malaria is associated with gametocytemia and results in false-positive diagnoses of Plasmodium vivax in convalescence J Clin Microbiol 2001 39 1025 1031 10.1128/JCM.39.3.1025-1031.2001 11230422 PMC87868

[B31] Baker J Ho MF Pelecanos A Gatton M Chen N Abdullah S Albertini A Ariey F Barnwell J Bell D Cunningham J Djalle D Echeverry DF Gamboa D Hii J Kyaw MP Luchavez J Membi C Menard D Murillo C Nhem S Ogutu B Onyor P Oyibo W Wang SQ McCarthy J Cheng Q Global sequence variation in the histidine-rich proteins 2 and 3 of *Plasmodium falciparum*: implications for the performance of malaria rapid diagnostic tests Malar J 2010 9 129 10.1186/1475-2875-9-129 20470441 PMC2893195

[B32] Gillet P Mori M Van Esbroeck M Van den Ende J Jacobs J Assessment of the prozone effect in malaria rapid diagnostic tests Malar J 2009 8 271 10.1186/1475-2875-8-271 19948018 PMC2789093

[B33] Endeshaw T Graves PM Shargie EB Gebre T Ayele B Yohannes G Zerihun M Genet A Melak B Kebede A Jima D Tadesse Z Ngondi J Mosher AW Richards FO Emerson PM Comparison of Parascreen Pan/Pf, Paracheck Pf and light microscopy for detection of malaria among febrile patients, Northwest Ethiopia Trans R Soc Trop Med Hyg 2010 104 467 474 10.1016/j.trstmh.2010.03.003 20378137

[B34] Seidahmed OM Mohamedein MM Elsir AA Ali FT el Malik FM Ahmed ES End-user errors in applying two malaria rapid diagnostic tests in a remote area of Sudan Am J Trop Med Hyg 2008 13 406 409 10.1111/j.1365-3156.2008.02015.x 18298604

[B35] Guthmann JP Ruiz A Priotto G Kiguli J Bonte L Legros D Validity, reliability and ease of use in the field of five rapid tests for the diagnosis of *Plasmodium falciparum *malaria in Uganda Trans R Soc Trop Med Hyg 2002 96 254 257 10.1016/S0035-9203(02)90091-X 12174772

[B36] Rennie W Phetsouvanh R Lupisan S Vanisaveth V Hongvanthong B Phompida S Alday P Fulache M Lumagui R Jorgensen P Bell D Harvey S Minimising human error in malaria rapid diagnosis: clarity of written instructions and health worker performance Trans R Soc Trop Med Hyg 2007 101 9 18 10.1016/j.trstmh.2006.03.011 17049572

