# Supplementary material for: Performance of three multi-species rapid diagnostic tests for diagnosis of Plasmodium falciparum and Plasmodium vivax malaria in Oromia Regional State, Ethiopia
Source: Malar J. 2010 Oct 27;9:297. doi: 10.1186/1475-2875-9-297 (PMC2988036; doi:10.1186/1475-2875-9-297)
Supplement: Additional file 1 — Description of the Ethiopian health system. [file 1475-2875-9-297-S1.DOC]

**The Ethiopian health system**

Ethiopia has a four tier health system: the lowest tier (primary health care unit) consists of a health center and satellite health posts. Health posts are staffed by salaried health extension workers who deliver a package of basic curative and preventative services, working both at the health post and in the community. Health posts have no laboratory services, but currently use RDTs for *P. falciparum* diagnosis and have access to ACTs for malaria treatment. Health centers exist at woreda (town) level and have laboratory facilities as well as providing limited inpatient services. Health centers routinely diagnose malaria by light microscopy including species identification, and are able to treat uncomplicated and severe malaria. The three higher tiers are district hospital, zonal hospital and specialized referral hospital.
