# Supplementary material for: Performance of three multi-species rapid diagnostic tests for diagnosis of Plasmodium falciparum and Plasmodium vivax malaria in Oromia Regional State, Ethiopia
Source: Malar J. 2010 Oct 27;9:297. doi: 10.1186/1475-2875-9-297 (PMC2988036; doi:10.1186/1475-2875-9-297)
Supplement: Additional file 2 — Semi-structured questionnaire used to evaluate RDT ease of use by health extension workers. [file 1475-2875-9-297-S2.DOC]

| **HEALTH POST DETAILS** | |
| --- | --- |
| **Health post name:** | **Health post code: |____|____|** |
| **Woreda:** | **Kebele:** |
| **HEW name:** | **HEW code: |____|____|____|** |
| **Date of interview (G.C.):** | **Name of interviewer:** |
| **RDT USED** | |
| **RDT in use at health post:** (circle one) ICT Combo ParaScreen CareStart | |
| **Duration of use:**  |____|____| months, *if less than 1 month:* |____|____| days | |
| **Has the HEW used any other RDT before?** (both during this study and as part of routine health post activities)  RDT 1 name: ________________________________ Length of time used:__________________________  RDT 2 name: ________________________________ Length of time used:__________________________  RDT 3 name: ________________________________ Length of time used:__________________________  RDT 4 name: ________________________________ Length of time used:__________________________ | |
| **RDT RATING:**  ***1 = very difficult to use, 5 = very easy to use*** | |
| **Format 1 2 3 4 5** | |
| **Lancet 1 2 3 4 5** | |
| **Swab 1 2 3 4 5** | |
| **Ease of writing details on device 1 2 3 4 5** | |
| **Ease of filling blood collection device 1 2 3 4 5** | |
| **Ease of emptying blood collection device 1 2 3 4 5** | |
| **Buffer drops 1 2 3 4 5** | |
| **Number of steps to carry out RDT 1 2 3 4 5** | |
| **Time to wait for results** (1=too long, 5=good length of time) **1 2 3 4 5** | |
| **Ease of interpreting test results 1 2 3 4 5** | |
| **Instruction leaflet 1 2 3 4 5** | |

| **FURTHER QUESTIONS** |
| --- |
| **In your opinion, how does this RDT compare with other brands of RDT you have used previously?**  (Begin by asking for comparison to ParaCheck, then to any other multi-species test used previously in this study) |
| **Additional comments** (probe for specific difficulties with the RDT, or favourable characteristics) |

| **OBSERVATION OF RDT PROCEDURE** |
| --- |
| **Observe the HEW carrying out an RDT test, to ensure correct procedure is followed.**  **Check for:**  Correct storage of RDTs  Correct labelling of RDTs  Fingerprick procedure  Blood collection using device in RDT kit  Emptying of blood collection device  Addition of correct buffer quantity  Waiting correct time before reading results  Interpretation of results  Recording of results on form |
| **Other comments, or specific difficulties observed:** |
